# Reconstruction of Large Upper Eyelid Defect with Two Composite Lid Margin Grafts

**DOI:** 10.4103/0974-9233.63083

**Published:** 2010

**Authors:** Amr Hafez

**Affiliations:** From the Research Institute of Ophthalmology, Giza, Egypt

**Keywords:** Upper Lid Reconstruction, Composite Eyelid Grafts, Lid Tumors, Lid Margin Defect

## Abstract

**Purpose:**

To evaluate the results of reconstruction of large defects in the upper lid using two composite contra-lateral eyelid margin grafts.

**Methods:**

This is an interventional case series in which a large full thickness defect of the upper lid was reconstructed in 7 patients using two composite eyelid margin grafts and myocutaneous advancement. The grafts were taken from both eyelids of the contra lateral eye and the final outcome was evaluated.

**Results:**

Cosmetic results were achieved in both donor and recipient eyes satisfying to all patients; no graft sloughing was seen. Transient edema, punctate epithelial erosions, lid notching, madarosis and ptosis were seen in the early postoperative period.

**Conclusion:**

Reconstruction of a large defect in the upper lid with two composite eyelid-margin grafts is an easy, safe, effective technique with minimal complications.

## INTRODUCTION

Reconstruction of an upper eyelid defect involving its margin, after tumor excision or trauma, is not an uncommon procedure. It offers special challenges because of the importance of its cosmetic appearance and diversity in ways of reconstruction.^1^–^6^ Application of proper technique will obviate most postoperative problems like eyelid notching, entropion, ectropion, trichiasis, ptosis and lagophthalmos.^7^ Due to the complexity of the detailed anatomy, upper eyelid defects are classically classified as anterior lamellar, posterior lamellar and full thickness defects. Several methods for reconstruction of the latter defect, such as direct closure with or without cantholysis, semicircular flaps, sliding tarsoconjunctival flap, pedicle flap from lower lid (Hughes procedure) and Cutler-Beard procedure are described. The choice of the procedure depends on the site and extent of defect. A patient with a large (more than 60% of lid length) full thickness defect of the upper lid and an excess skin of the upper lid is our concern in this study and a good candidate for this technique. A composite eyelid margin graft taken from a healthy lid should not exceed one third of the lid margin length to allow for a direct closure of the induced defect with good functional and cosmetic results.^8^ For other surgeons the maximum length of donor lid that can be taken from a normal eyelid has been recommended as 8 mm.^9^ Very large defects are classically not suitable for composite eyelid margin grafts.

The study takes two composite eyelid margin grafts from the eyelids of the sound eye to close the large defect in the upper eyelid in the affected eye.

## MATERIALS AND METHODS

In this interventional case series, seven patients with an upper lid tumor (basal cell carcinoma) were operated upon by one surgeon. The lesions were excised and reconstruction of the resulting lid defect performed. Details of surgery were explained to all patients and an informed consent was obtained prior to the intervention. A full ophthalmological and medical evaluation was done. These patients were selected as they met the following criteria after excising of the tumor with a safety margin:

1. Large upper eyelid defect that exceeded 60% of lid margin length.
2. Good amount of remaining skin of the upper eyelid of involved eye.
3. Presence of residual nasal and temporal parts of tarsus or at least portion of the medial and lateral palpebral ligaments.
4. Normal eyelids in contra lateral eye.

### Technique

**Figure 1:**
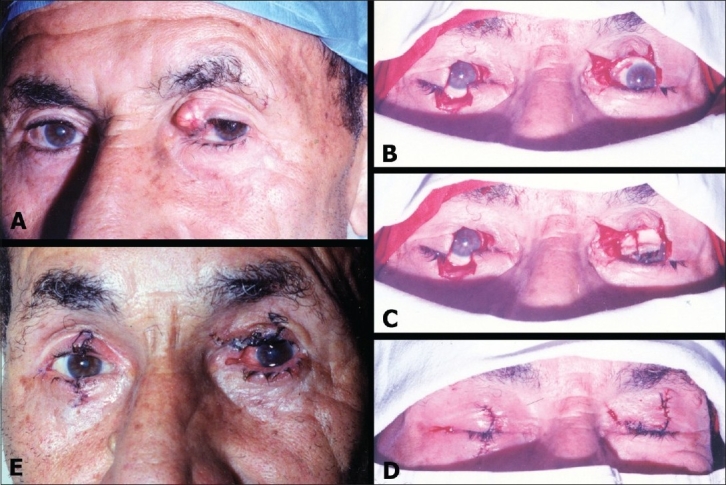
A- Left upper lid tumor. B- Lid defects after excision of the tumor (left eye) and the two pentagons (right eye). C- Two composite grafts in place. D- Lids reconstructed. E- Fourth postoperative day with sutures not yet removed

Reconstruction of the upper eyelid was done under local infiltration anesthesia to both eyes. The procedure was initiated by harvesting two composite marginal grafts, one from the upper eyelid and the other from the lower eyelid of the sound eye [Figure 1]. Each of these grafts was obtained by excising a pentagon from the middle third of the eyelid. The size of each pentagon was not more than 30% of the lid margin length to allow repair of resulting defects by direct closure. The grafts were harvested by removing the skin and orbicularis of the excised pentagons leaving about 2.5 mm of skin intact distally to preserve the eye lashes. The resulting pentagon defects are closed by direct suturing as described by Collin.^8^

### Reconstruction of posterior lamella

The two composite eyelid margin grafts were used to create the new posterior lamella of the defect. The grafts were placed to fill the lid defect with their conjunctival surface lying on the globe. The graft from the lower eyelid was rotated so that the lid margin faced downwards forming a continuous upper lid margin configuration. The composite graft from the lower eyelid was smaller than the one from the upper eyelid as the lower eyelid tarsus is smaller than the upper. Placing the smaller composite graft in the nasal or temporal site of the defect was arbitrary according to the need for proper construction of the posterior lamella and creating a good lid margin contour. The nasally placed composite graft was fixed first to the nasal part of the tarsus (or to the medial palpebral ligament) and sutured as in direct closure of lid margin wound, taking care to place these sutures intratarsally to prevent their rubbing against the cornea. Next, the second composite eyelid margin graft is fixed between the first one and the temporal remains of the original tarsus of the eyelid in the same manner. A lateral cantholysis could be done according to need to prevent any overstretch on the repaired eyelid. The upper parts of these composite eyelid margin grafts were sutured using interrupted 6-0 vicryl to remaining tarsus or to the conjunctiva and the levator palpebri muscle after adjusting its vertical height to maintain a good lid contour and good levator function.

### Reconstruction of anterior lamella

Skin and orbicularis muscle above the defect in the upper eyelid were dissected as needed and mobilized to cover the new tarsus in the form of a myocutanuous advancement flap and sutured in a single layer to the skin of the new lid margin and anterior surface of the tarsus using 6-0 vicryl sutures. Redundant Burrow's triangles, if present, were excised to prevent dog ears.

## RESULTS

The patients (five males and two females) had a mean age 65 ± 6.1 years (range 56-75 years old). The right eyelid was involved in four patients and left eyelid in three patients. The lid defects in all patients were secondary to tumor excision. The size of the defect ranged from 13 to 20 mm. The average follow-up period was 11 months (range 3-25 months). All patients were satisfied from the cosmetic outcome. Mild lid edema was seen in three patients during the first one to three postoperative weeks. Moderate lid edema with ecchymosis was seen in one patient. The lid edema subsided in all patients Within few weeks from surgery. Ptosis of the reconstructed upper eyelid is seen in two patients (one of them with ecchymosis). The ptosis disappeared within four weeks in one patient and became minimal in the other (the patient with ecchymosis) where 1.5mm residual ptosis was noted. The patient was satisfied by his appearance and refused any further intervention. All patients were found to have an aligned lid margin. A small lid notch was seen in patients whose composite graft was sutured to the palpebral ligament directly without any tarsal remnants. Punctate epithelial keratopathy was detected in two patients. Two patients developed an area of madarosis at the newly constructed lid margin. No postoperative graft sloughing occurred though the use of two side by side composite grafts. Neither entropion nor lagophthalmos had occurred. In only two of the seven patients a lateral cantholysis was needed. No free skin grafting was used in any patient. The results are summarized in Table 1

**Table 1:** Summary of surgical results

Patient	1	2	3	4	5	6	7
Size of defect	14 mm	13 mm	15 mm	20 mm	14 mm	13 mm	16 mm
lid edema	mild	No	mild	moderate	no	mild	no
Ecchimosis	no	No	no	yes	no	no	no
Ptosis	temporary	No	no	Permanent 1.5 mm	no	no	no
Lid margin	good	Good	Small notch	good	good	good	Small notch
Punctate erosion	no	yes	no	no	yes	no	no
madarosis	no	yes	no	yes	no	no	no
lagophthalmos	no	no	no	no	no	no	no
entropion	no	no	no	no	no	no	no
Lateral cantholysis	yes	no	no	yes	no	no	no
Graft sloughing	no	no	no	no	no	no	no
Skin grafting	no	no	no	no	no	no	no

## DISCUSSION

Upper eyelid reconstruction is a delicate procedure as it implies restoration of the normal lid contour, mobility, height and integrity of its margin and conjunctival surface. The diverse reconstruction techniques suggest that none of the procedures provide a perfect outcome. A composite tarso-marginal graft is now considered an important method for lid reconstruction.^10^ In some surgical approaches, as in Hughes procedure,^5^ Cutler-Beard procedure^1^ and skin mobilization with posterior lamellar graft,^8^ reconstruction of the lid margin is done by suturing the conjunctiva to the skin of the lid. Other approaches used to repair smaller defects result in an almost normal lid margin as in direct closure, Tenzel semicircular flaps^3^ and composite grafts.^11^

The technique described in this paper forms a normal lid margin with its transition zone and lashes resulting in better cosmesis. The use of two composite marginal grafts, instead of one, allows a larger graft size and hence permitting the repair of a larger defect without horizontal tension. In other words, this technique combines the benefits of repairing an upper lid defect of considerable length and height while keeping a good architecture of the lid margin in a single step procedure. As a result, the incidence of postoperative entropion and ectropion, common in other procedures,^12^ is not seen with this procedure. The original description of using a double composite graft for upper lid reconstruction was reported by Beyer *et al.*^13^ but they used a rotational pedicle advancement skin flap and a free skin graft to cover the defect created by such a flap.

In this study, a small modification of the technique described by Beyer was used. The modification was that, no free skin grafts were used because the patients had excess skin of the upper eyelid to cover the raw area by an advancement flap. Proper suturing of the lid margin did not cause any lid notching except in a case where the nasal composite graft is sutured directly to the medial palpebral ligament without any remaining host tarsus. The appearance is cosmetically good without any functional deficit. The reason for developing ptosis in one patient was not the horizontal tension on the lid but a rather vertically large graft size. A one-stage procedure is also an advantage to be added to this technique as compared to others^1^,^5^ where the patient has to cover his eye for a longer time. The disadvantage of this technique is multiplicity of the operative sites, where both lids of the sound eye in addition to the defect site are involved but excellent results of reconstruction by direct lid closure is evidence that procedure is effective. Free skin grafting was not required as the skin of the upper eyelid in older individuals is loose and provides sufficient tissue to stretch. Lagophthalmos was not seen as a complication of this technique as there is ample tissue in the composite grafts that prevents lid shortening. The technique described offers a one-stage procedure with a simple surgical technique providing a good cosmetic and functional result utilizing two composite eyelid-margin grafts and local advancement myocutanuous flap.

## CONCLUSION

Reconstruction of a large defect in the upper lid with two composite eyelid-margin grafts is a one step, easy, safe, effective technique with minimal complications. It also has the advantage of restoration of an almost normal lid margin.
